# Inadequate Status and Low Awareness of Folate in Switzerland—A Call to Strengthen Public Health Measures to Ensure Sufficient Intakes

**DOI:** 10.3390/nu12123729

**Published:** 2020-12-03

**Authors:** Isabelle Herter-Aeberli, Nina Wehrli, Kurt Bärlocher, Maria Andersson, Janice Sych

**Affiliations:** 1 Human Nutrition Laboratory, Department of Health Sciences and Technology, ETH Zurich, 8092 Zurich, Switzerland; nina.wehrli@alumni.ethz.ch; 2 Scientific Board of the Folic Acid Foundation Switzerland, 6302 Zug, Switzerland; k.baerlocher@bluewin.ch; 3 Nutrition Research Unit, Children’s Research Center, University Children’s Hospital Zurich, 8032 Zurich, Switzerland; maria.andersson@kispi.uzh.ch; 4 Institute of Food and Beverage Innovation, ZHAW School of Life Sciences and Facility Management, Einsiedlerstrasse 34, 8820 Waedenswil, Switzerland; sych@zhaw.ch

**Keywords:** folate status, folic acid, women, pregnancy, folate awareness, neural tube defects

## Abstract

Background: Folate plays an essential role in the prevention of neural tube defects, yet little is known about the folate status of women of reproductive age or to what degree the general population is aware of the importance of folate in early-life development. We aimed to determine folate status in women of reproductive age and pregnant women in Switzerland, and to assess folate awareness in the Swiss population. Methods: In a convenience sample of 171 women of reproductive age and 177 pregnant women throughout Switzerland, we measured red blood cell (RBC) folate concentration. In a second convenience sample (*n* = 784, men and women) we assessed folate knowledge with an online survey. Results: RBC folate concentration (median interquartile range) was 442 (366, 564) nmol/L in women of reproductive age and 873 (677, 1177) nmol/L in pregnant women. Folate deficiency (RBC folate <340 nmol/L) was found in 19.9% of women of reproductive age and 2.8% of pregnant women, while 91.8% of women of reproductive age and 52.0% of pregnant women showed folate concentrations indicating an elevated risk of neural tube defects (RBC folate <906 nmol/L). The online survey showed that a high proportion (≥88%) of participants were aware of folate’s role in neural tube defect (NTD) prevention and fetal development, yet knowledge about dietary sources and national recommendations of folate supplementation when planning pregnancy were limited. Conclusion: The high prevalence of folate inadequacy in Swiss women suggests an elevated risk of neural tube defects and calls for urgent measures to increase folate intakes.

## 1. Introduction

It is widely acknowledged that adequate folate status during the periconceptional period is critical and folic acid supplementation before conception helps to substantially reduce the risk for neural tube defects (NTDs) [1]. Based on this reduction in risk, most health organizations recommend that women of reproductive age (WRA) and especially those planning a pregnancy take daily folic acid supplements at levels of 400 µg or higher (folic acid or methyl folate), beginning at least four weeks before conception and continuing throughout the first 12 weeks of pregnancy [2,3]. In combination with a folate-rich diet [4,5], this recommendation should allow women to reach optimal red blood cell (RBC)–folate levels which are associated with NTD risk reduction, recently defined as >906 nmol/L [6]. However, several factors may impact the time required to reach this target, such as low folate status due to low dietary folate intake and differences in biological responses to folic acid supplements [7,8]. Despite convincing evidence for NTD preventive effects associated with folic acid, a major problem is low adherence to the folic acid supplement recommendation, as reported in many countries [5,9]. This is partly caused by unplanned pregnancies, but also by overall low awareness of folate/folic acid and its role in the correct formation of the neural tube of the fetus. This development occurs during the first 28 days of pregnancy, consequently the correct timing of supplements is a critical aspect of the recommendation but is particularly challenging to communicate to the general public. Consequently, NTDs are still considered a major, preventable public-health burden which resulted in approximately 50,000 deaths in 2016 [10].

Mandatory fortification of staple foods with folic acid is another proven intervention to increase the folate intake in the general population and decrease the incidence of NTDs [11,12]. This typically provides an additional 100–200 μg folic acid/day to WRA [13]. Globally, 84 countries had mandatory fortification in 2019 [14,15]. However, an estimated 215,000 NTD-affected pregnancies still occurred as a result of inadequate or nonexistent fortification policies [11]. In Switzerland, the prevalence of NTDs is comparable to other European countries and lower than the global estimates [16,17,18]. Furthermore, the NTD incidence remained stable between 1989 and 2017 [16]. Pre- and peri-conceptional folic acid supplementation is the main recommended public health measure in Switzerland, which is complemented by the availability of folic acid fortified food products (on a voluntary basis) [19].

In the first national survey of folate status conducted in pregnant women (PW) in Switzerland (2001, *n* = 381), 63% of women answered that they took a supplement of folic acid, but women were not asked when they started taking the supplements. Low serum folate (<2.5 µg/L) was reported in 4% of the women, and the concentration was significantly higher in the second and third trimester of pregnancy in folic acid supplemented compared with un-supplemented women [20]. In the German-language region (in the years 2002–2003), folate status and knowledge in women (*n* = 598) assessed in the postpartal period showed high (60–100%) overall awareness of the importance of folate in early life development [21]. German-speaking women were well informed about the correct timing of supplement intake (ca. 75%), while women with an eastern European background were less informed (24%). Almost all PW (97.5%) took a supplement of folic acid, but only 32% had started the supplements four weeks prior to conception. Postpartal red blood cell folate concentration was >906 nmol/L in 82% of respondents [21] and no child was born with NTD during the study.

In a later survey (2009) conducted in over 22,000 women in 18 European countries including Switzerland, 70% of respondents (77% in Switzerland) had heard of folate or folic acid but only 17% (33% in Switzerland) were aware of its preventive effects against NTDs. Furthermore 38% of all first pregnancies amongst respondents (27% in Switzerland) were unplanned and in 75% of planned pregnancies (ca. 80% in Switzerland), women did not talk to a doctor before stopping contraception [9]. The data show a lack of awareness of folate in the population, especially in WRA. More data on information sources and channels are needed for targeted awareness campaigns.

In this study, we aimed to: (1) assess folate red blood cell concentration in WRA and PW in Switzerland; and (2) investigate the awareness of the importance of dietary folate and pre- and peri-conceptional folate supplementation in the general Swiss population.

## 2. Methods

### 2.1. Folate Status in WRA and PW in Switzerland

#### 2.1.1. Study Design

This study was conducted in the framework of the Swiss Iodine Survey 2015. Detailed methodology of the original study is described elsewhere [22]. Briefly, we conducted a cross–sectional study in April 2015–January 2016 and aimed to obtain a representative national sample of WRA and PW. For the current analysis, only women who gave venous blood samples were included. We contacted 346 obstetric/prenatal care clinics/hospitals throughout Switzerland by e-mail and 29 clinics/hospitals agreed to participate. Of those, eleven withdrew again without enrolling any participants. Of the 18 remaining clinics/hospitals, nine collected venous blood samples for folate analysis in addition to the samples collected for the iodine survey.

#### 2.1.2. Participants

The inclusion criteria for both WRA and PW were: (1) residence in Switzerland for ≥ 12 months, (2) general good health (assessed by no reported treatment for chronic disease, gastrointestinal or metabolic disorders), (3) no family history of thyroid disease, (4) no exposure to iodine-containing contrast agent or medication within the last year. WRA were non-pregnant and non-lactating and PW had a healthy, singleton pregnancy.

The original sample size calculation was done in relation to iodine status. The sample of the current study can be described as a convenience sample of volunteers within the larger study.

We obtained ethical permission for the study from the Ethics Committee of the Canton Zurich (KEK–ZH Nr. 2014–0692), and written informed consent from all participating women. All data were collected in a coded way and registered only by participant number, location and age. The study was registered at ClinicalTrials.gov as NCT02312466.

#### 2.1.3. Study Procedures

Study participants were recruited by the supervising physician in the participating obstetric/prenatal care clinics. We informed the physicians about the study procedures and they instructed the participants. Eligible women were provided with the written study information and were given sufficient time to read and take a decision. Written informed consent was obtained before sampling was initiated. The participants received no compensation for their participation.

A short questionnaire was administered to assess inclusion and exclusion criteria as well as basic information on diet, health, and socioeconomic status. Venous blood samples were collected in EDTA-coated vacutainers. Folate in erythrocytes (RBC folate) was determined from whole blood using Abbott Architect i2000 using a chemiluminescence immunoassay with magnetic particles (Abbott Laboratories, Wiesbaden, Germany). Folate deficiency was defined as RBC folate <340 nmol/L and a folate concentration indicating elevated risk for neural tube defects (hereafter referred to as folate insufficiency) was defined as RBC folate <906 nmol/L) based on WHO recommendations and according to the manufacturer of the laboratory test (Abbot Laboratories) [6,23].

### 2.2. Folate Knowledge in the Swiss Population

#### 2.2.1. Questionnaire

We developed an online survey in German and English using the software “Unipark” (Table S1, Supplementary Materials). The questions were selected based on a literature research on studies regarding folate awareness and inputs from experts from the scientific board of the “Stiftung Folsäure Schweiz”, a Swiss foundation which aims to better inform the public about the importance of folate. The questions were arranged in five different topic categories: (1) current knowledge on folate; (2) attitude and interest regarding nutrition and health in general (used to define nutrition consciousness); (3) sources of information regarding nutrition and health; (4) demographics; and (5) female-specific questions. All questions were multiple choice. The survey could be accessed using a weblink or a QR code. To screen for errors and ambiguity, the questionnaire was pre-tested in a group of 12 adults aged between 26 and 42 years, from the general public and with varying backgrounds.

#### 2.2.2. Study Procedures

We aimed to distribute the questionnaire as widely as possible among the general public. To achieve this goal, we recruited participants by the following strategies: (1) we asked 129 general physicians across the German speaking part of Switzerland to display the study flyer including QR code and link in their waiting rooms. Of those, 41 replied, and 18 agreed to display the flyer. (2) We distributed the study information including the link to the study via an e-mail distributor to students of the Department of Computer Sciences as well as the employees of the Department of Health Sciences and Technology at ETH Zurich. (3) The study authors shared the link in their private and work environment as well as on social media. The survey was open for participation in June and July 2019. Participation in the survey was anonymous, no contact information was collected from the participants.

### 2.3. Statistical Analysis

All data were analyzed using the software IBM SPSS Statistics Version 24 and Microsoft EXCEL 2016. Data on folate status are presented as median with interquartile range (IQR) as well as percentage. Differences in folate status between trimesters of pregnancy were calculated using Kruskal–Wallis test with post-hoc Bonferroni correction. Differences between women taking and not taking folate supplements were tested using Mann–Whitney U test. In the survey, only respondents who completed the survey were included in the analysis. After data cleaning, differences in answers were assessed across the population groups (sex, age, nationality, education, income, and nutrition consciousness) using individual chi–square tests. Statistical significance was set at *p* < 0.05 for all analysis.

## 3. Results

### 3.1. Folate Status in WRA and PW in Switzerland

Blood samples were collected from 171 WRA and from 177 PW (*n* = 20, 78, and 79 in the first, second, and third trimester, respectively). The results of the red blood cell (RBC) folate concentration are shown in Table 1. The prevalence of folate deficiency (<340 nmol/L) was 19.9% in WRA and 2.8% in PW. The prevalence of RBC concentrations indicating elevated risk for neural tube defects (<906 nmol/L) was considerably higher at 91.8% in WRA and 52.0% in PW. In PW, we observed the highest RBC concentration in women in their third trimester of pregnancy (994 nmol/L) and the lowest folate concentration in the first trimester (713 nmol/L, *p* = 0.002 first vs. third trimester). The proportion of RBC folate <906 nmol/L was 75% in the first trimester, 47% in the second trimester and 56% in the third trimester. Based on the questionnaire administered to all women, 19 out of the 171 WRA (11%) and 146 of the 177 PW (83%) consumed supplements containing folate at the time of sample collection. No information was available on the starting time and duration of supplement intake. RBC folate concentration was significantly higher in women consuming supplements compared to those who did not (935 vs. 631 nmol/L in PW (*p* = 0.002), and 720 nmol/L vs. 420 nmol/L in WRA (*p* < 0.001).

### 3.2. Folate Knowledge in the Swiss Population

A total of 937 participants accessed the survey and 784 completed questionnaires (completion rate: 83.7%). Of these respondents, 482 were female and 302 were male, with an average age of 34.4 ± 12.2 years. Characteristics of the participant are shown in Table 2. Eighty one percent of participants reported an education at the tertiary level, which is almost double compared to the general Swiss population aged between 25 and 64 years (44%) [24]. The highest percentage of participants (67%) was in the highest income category.

Category 1 of the questionnaire, current knowledge on folate, was assessed using four questions. In total, 61% of respondents (66% of women and 52% of men) correctly identified folate as an important vitamin when asked “What is folate?”. A significantly higher proportion of women, participants aged 35 years or older, those with a tertiary degree, and those with a high level of nutritional awareness answered correctly (all *p* < 0.001), while nationality (*p* = 0.487) and income (*p* = 0.603) had no effect on the answer.

**Figure 1:**
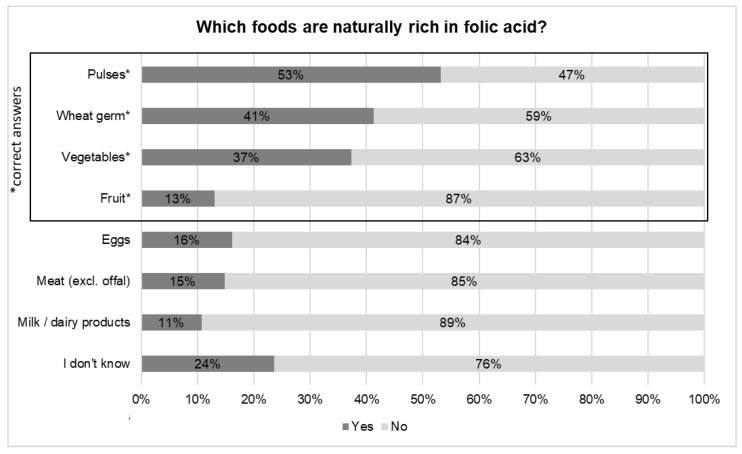
Identification of food sources naturally rich in folate in a survey on folate knowledge in the Swiss population (*n* = 784). * Indicates the correct answers.

In contrast, the question on natural sources of folate in food was answered correctly by only 2% of respondents. Nevertheless, 37%, 41% and 53% of respondents correctly identified vegetables, wheat germ, and pulses, respectively, as foods rich in folate, while only 13% were aware that fruits are good folate sources (Figure 1). In total, 74% of respondents correctly identified at least one natural source of folate, and women more often correctly identified the sources.

The third question asked about beneficial effects of folate. Of all respondents, 88% and 93% identified a positive effect regarding risk reduction of spina bifida and the normal development of the fetus in the uterus, respectively. Concerning those two areas of knowledge, females, older respondents (≥35 years), those with a tertiary degree and high level of nutritional awareness correctly identified a benefit of folate more often (all *p* < 0.001, except age effect in the question on normal fetal development: *p* = 0.012). A total of 34% of respondents correctly identified a positive effect of folate on sperm amount and quality, and participants of male sex (*p* = 0.009), younger age (<35 years, *p* < 0.001), and with no tertiary degree (*p* = 0.043) correctly selected this effect more often. On the other hand, a majority of participants knew that folate has no beneficial effect on dental health (80%) or vitamin C absorption (62%).

**Figure 2:**
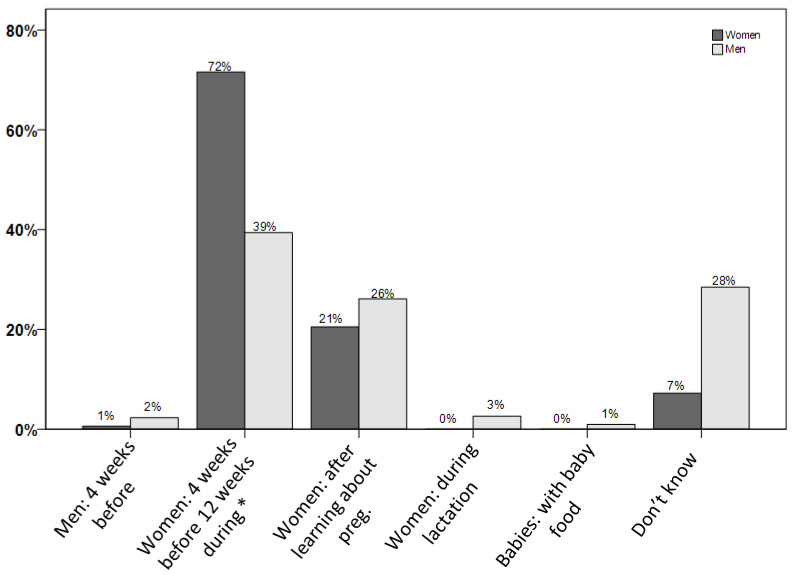
Knowledge on the recommended timing of folate supplementation in a survey on folate knowledge in the Swiss population (*n* = 784). * Indicates the correct answer.

The final question in this part of the questionnaire was on the recommendation of folate supplementation to reduce the risk for spina bifida. Of all respondents, 59% correctly answered this question, with a higher proportion of women (72%) compared to men (39%) (*p* < 0.001, Figure 2). Study participants with higher proportions of correct answers were aged above 35 years (*p* = 0.005), had a higher education (*p* < 0.001), a higher income (*p* = 0.01), and higher nutritional awareness (*p* < 0.001).

Category 3 of the questionnaire asked about attitude and interest regarding nutrition and health in general. When asked about main source of folate knowledge, the answers were widely distributed: 19% from school, 15% from doctors or medical professionals, 15% from friends or family, 13% from media, 10% from specialist literature, and the remaining 4% from pharmacy or other sectors. A further 24% answered to have no knowledge about folate, with a much higher proportion of men (41%) compared to women (14%).

When we asked participants about their preferred source of information on folate, media (*n* = 343) and doctors/medical professionals (*n* = 332) scored highest (as multiple answers were possible, this is not given in % and the total n exceeds the number of participants of 784). This was followed by specialist literature (*n* = 291), school (*n* = 260), pharmacy (*n* = 174), and friends/family (*n* = 131). As sources of folate information, women had higher preferences for doctors and pharmacies compared to men, however participants aged <35 years had lower preferences for doctors but higher preferences for schools. Participants without a tertiary degree and those with lower income relied more on schools for their information, while those with a tertiary degree preferred specialized literature.

The last few questions (category 5) were asked to female participants only. The majority of women (86%) confirmed regular visits to the gynecologist for a check-up yearly or every second year. However, frequency of gynecological check-ups was lower for women in the lower income class compared to those in the higher income class (*p* < 0.001). Only 38% of all women recalled having received information regarding the importance of folate in planning a pregnancy during those regular check-ups.

**Figure 3:**
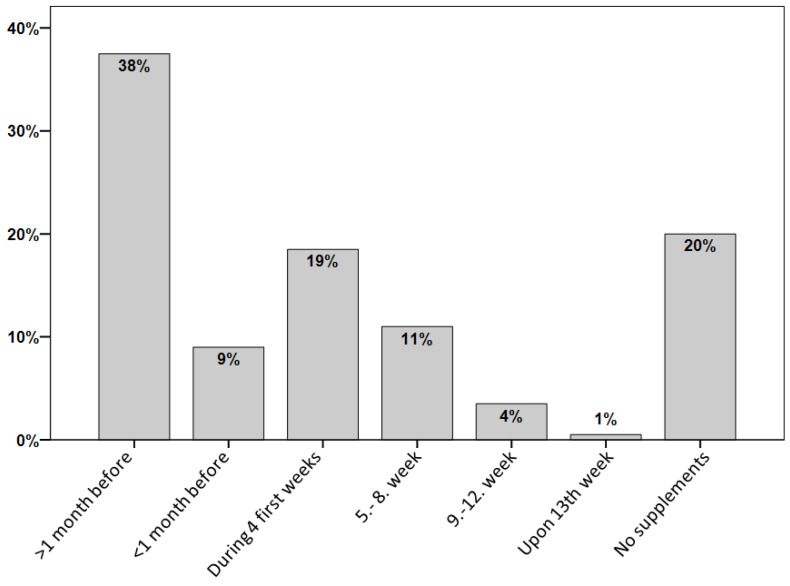
Timing of folate supplementation as reported by female participants of a survey on folate knowledge in the Swiss population who were pregnant at the time of the survey or already had children (*n* = 200).

Of all PW and women who already have children (*n* = 200), 38% started taking folate supplements at least one month before conception. However, 20% of the women did not take any folate supplements and 44% started taking them at a later point than the recommended time, i.e., one month prior to conception (Figure 3). Education was an important predictor of the timing of supplements in association with a pregnancy as shown by 41% of women with tertiary degree who reported taking supplements ≥1 month prior to conception compared to only 16% of women without a tertiary degree (*p* = 0.017). Similarly, women in the higher income class were more likely to take the supplements at the recommended time (43%) compared to those in the lower income class (21%) (*p* = 0.014).

In the same group of women, while 29% of the participants did not receive any information about nutrition during pregnancy by their gynecologist, 64% received this information during pregnancy, and 7% received this prior to their pregnancy. Women reported that they were informed about folate deficiency and the risk of birth defects by their gynecologist before pregnancy (25%) and during pregnancy (21%), whereas 54% were not informed at all. Similarly, regarding the relationship between folate and spina bifida and the importance of taking supplements, 39% and 42%, respectively, were informed prior to pregnancy, and 31% and 35% were informed during pregnancy. On the other hand, 30% and 23%, respectively, were never provided with any information about this relationship and the importance of folic acid supplements.

## 4. Discussion

Periconceptional folate supplementation, in addition to a balanced diet, has consistently been shown to significantly reduce the NTD risk [1,25]. To our knowledge, this is the first study to report data on folate status in WRA in Switzerland and data on folate awareness in the general Swiss population, leading to the following critical discussion of the overall folate landscape.

RBC folate concentrations at the population level should be >906 nmol/L in order to achieve the greatest reduction in NTDs [6]. In our convenience sample of WRA and PW, we identified a high proportion of WRA (91.8%) and PW (52.0%) with RBC folate concentrations <906 nmol/L. In the first trimester, only one in four PW had RBC folate concentrations >906 nmol/L. Our results confirm previous findings of low dietary folate intake [26]. This earlier study used 10-day weighed-food records of 25–35-year-old single women in the Zürich area and reported a daily folate intake of 122 µg, which is considerably below the DACH recommended daily intake of 300 µg/day [5]. The low folate status observed in our study may be related to the low proportion (11%) of WRA consuming dietary supplements containing folate. This proportion increased to 83% in PW, but a delayed start of supplement intake may explain the high proportion of folate insufficiency in the first trimester. Our data also clearly showed significant differences in RBC folate concentrations in women taking folate supplements compared to those who did not, which accentuates the direct effect of those supplements.

Folate status biomarkers and thresholds often differ between studies, complicating the comparison of folate status between countries [20]. Additionally, previous studies on folate status in Switzerland are limited. However, we observed higher prevalence of RBC <906 nmol/L in PW (52%) than that reported in a regional study of women just after giving birth (18%) [21]. In our study, the prevalence of folate insufficiency was highest in the first trimester, decreasing throughout pregnancy and the late start of folate supplementation may explain the discrepancy with previously reported postpartum folate status. Lower prevalence of folate insufficiency (assay-specific cut-off of <748 nmol/L, which is equivalent to the WHO cut-off of 906 nmol/L) of 18.6% has been reported in 12–49-year-old women in the US [27], where mandatory folate fortification of enriched cereal grain products has been in place since 1998 [28]. A German study conducted between 2008 and 2011 reported that 86% of the adult population, and 88.2% of the female population, was sufficiently supplied with folate [29]. However, this estimation was based on serum folate concentrations >10 nmol/L, which is considered as the cut-off for possible deficiency on the basis of metabolic indicators (elevated homocysteine, RBC cut-off of 340 nmol/L) and not for protection against the NTD risks [6]. These results are comparable to our findings, which indicate sufficiency based on a cut-off of RBC folate >340 nmol/L for 80.1% of WRA and for 97.2% of pregnant women.

It is estimated, that mandatory folic acid fortification has prevented >1300 NTDs every year in the US [30], yet the practice is still highly debated [31,32,33]. Concerns involve potential risk of exceeding the upper tolerable intake limit in vulnerable groups, possibly increasing the risk for: (1) unmetabolized folic acid; (2) potential epigenetic changes and increased cancer risk, and; (3) masking of diagnostic signs of vitamin B12 deficiency [31,32,34]. The UK has recently taken steps to introduce mandatory folate fortification of targeted staple foods, but there is still no consensus in Europe [35,36]. Switzerland has about 200 food products enriched with folic acid by the food industry on a voluntary basis. Consumption of fortified foods is highly dependent on public awareness of the importance of consuming such products. Important limitations of voluntary fortification are a lack of centralized control, variable coverage of the population, and limited traceability of overall intake of the targeted nutrient.

In Switzerland, national data on the prevalence of NTDs are scarce. Data from one canton (Vaud) are collected yearly and reported via the European platform EUROCAT [16]. These data show 10.2 cases per 10,000 births (this includes both live and still births as well as terminations of pregnancies after prenatal diagnosis of anomalies) between 1989 and 2017, of which only 1.9 were born alive. These estimations are comparable to a median of 9.0 cases per 10,000 births reported for all European countries [18]. Globally, the prevalence of NTDs ranged from 1.2 to 124.1 per 10,000 births, but data were often regional as national registries are limited. The study concluded that approximately 80% of estimates included reported NTD prevalence above 6.0 per 10,000 births [18]. Based on pre- and post-fortification data from countries with mandatory folic acid fortification of staple foods, Crider et al. concluded that the prevalence of NTDs could be reduced to five to six cases per 10,000 births and that a certain proportion of NTDs is not sensitive to folate [32]. In Europe, it is estimated, that around 2400 pregnancies develop folic acid-preventable NTDs per year [7]. Thus, the above data suggest a high potential to reduce the incidence of NTD and to avoid terminations of pregnancies due to NTD diagnosis by improving folate prophylaxis in Switzerland either by supplementation or fortification.

In the absence of mandatory folic acid fortification programs, folate status can be improved by targeted folate supplementation of 400 µg/day, but timing supplementation before conception is challenging [5,37,38]. Further, compliance is influenced by: age (lower adherence at younger age), education (lower with lower education), income (lower with low income), smoking before pregnancy (lower for women who smoke), weight status (lower in obesity), and immigration background (lower in immigrants) [7]. In our survey, we demonstrated that both knowledge about folate sources and the correct timing of supplementation was higher in women compared to men. For the correct time of supplementation, higher education and income as well as age above 35 years also appeared to be important predictors. Considering the greater representation of highly educated participants in our study compared to the general population, the awareness of the importance of folate supplementation during pregnancy may even be lower in the general population than that observed in our study. In addition, older participants with better folate knowledge could be a result of a higher proportion of them having already undergone or supported a partner or friend during a pregnancy. This may, however, also indicate that this knowledge is acquired too late for many of the survey participants, which emphasizes the importance of introducing this important information early in life.

Only 38% of the women participating in our survey who were currently pregnant or already have children indicated that they started folic acid supplementation at least four weeks prior to conception. This is in agreement with the high prevalence of folate insufficiency detected in our convenience sample of both WRA and PW and especially with our result of lower folate status of women in the first compared to the third trimester of their pregnancy. In the survey, only a minority of women (38%) had received information regarding the importance of folate before and during pregnancy from their gynecologist during regular check-ups, even though >85% of women did report having regular check-ups every one or two years. Our findings are in line with a large survey conducted in 18 European countries, including Switzerland (in 2009), where the awareness of the importance of folic acid in general and related to pregnancies was also found to be low [9]. Only 17% of the >22,000 respondents were aware that folic acid reduces the risk of NTDs and only 28% of those women currently trying to become pregnant were taking folic acid supplements [9]. A recent study from France revealed that only 6.4% of women filled their prescription for folate supplements in the preconceptional period between 2006 and 2016, based on objective data from registries [39]. Even though over-the-counter supplements were not included, this low number may indicate that self-reported adherence to supplement recommendations, as used in most other studies, might be overestimated to a certain extent. These results are consistent with our findings which emphasize the need for new approaches to increase awareness of folate’s critical role in early life development, with the ultimate goal of better adherence to the NTD preventive recommendation, both before and during pregnancy. Our findings suggest that regular visits to the gynecologist might be an important opportunity to better inform women on this topic, and also to emphasize the importance of nutrition before and during pregnancy [40]. Evidence suggests that women with very low folate status due to poor diet or other reasons might need to start folic acid supplements earlier than the recommended 4 weeks in order to reach the targeted RBC folate level (>906 nmol/L) [41]. As the correct timing of supplementation was strongly influenced by higher education and income, targeted information strategies adjusted for these factors might also be valuable. Large differences in folate awareness between men and women indicate that more efforts might be needed to inform men about NTD-preventive measures and the specific time-frame for folate supplementation, and continual efforts are needed to prioritize the role of folate intake both before and during pregnancy.

To our knowledge, this is the first study to compare measured folate concentrations in WRA with the results on folate knowledge derived from a survey using a separate study group comprised of men and women. Although the samples for folate status in WRA and PW were collected on a national level, they were not representative samples due to the limited response. Therefore, we cannot rule out a certain bias in our study population. A nationally representative study involving a larger sample size is warranted. For the online survey, our recruitment aim was to include as many participants as possible with varying socioeconomic backgrounds. However, to achieve the targeted numbers of participants, we had to resort to e-mail lists available in our University. By sending the e-mail to employees of the Department of Health Sciences and Technology of ETH Zurich, we hoped to include both people working directly in a health-related field, and all technical and administrative personnel. The choice of students from the Department of Computer Sciences at ETH Zurich was also intentional, as we expected those students to be less interested in health issues compared to some other study programs. Nevertheless, with the use of those two mailing lists, there was a resulting bias for a higher proportion of participants with higher education. In order to take this into account in the analysis, we examined the effect of education on the results whenever possible. Still, despite the high overall educational level of the study population in our survey, the distribution of participants in terms of income categories was similar to the National Nutrition Survey menuCH [42].

## 5. Conclusions

In conclusion, we report a high proportion of inadequate folate intake in both WRA and PW in Switzerland, along with low adherence to supplementation recommendations. Furthermore, the awareness of folate’s essential role before and during a pregnancy was low, especially in the younger adults and groups with low education level. These results highlight the need to strengthen public health strategies and foster better co-ordination for existing measures aimed to improve awareness and ensure adequate folate intake, especially in women planning pregnancy. New approaches might be needed to reach the younger population, such as the use of digital media or by introducing the topic at an earlier age through the school curriculum. There seems to be high potential for raising the awareness of folate requirements and nutrition during pregnancy at the routine check-ups with the gynecologist, which are attended by a majority of women. To facilitate reaching targeted periconceptional RBC folate levels, improvements in the present system of voluntary folic acid fortification might be achieved by increased control and monitoring, or by considering mandatory fortification of targeted staple foods.

## Figures and Tables

**Table 1:** Folate status and prevalence of folate deficiency in women of reproductive age and pregnant women in Switzerland.

	Women of Reproductive Age	Pregnant Women
*n*	171	177
Age (year) ^1^	31.4 (25.0, 36.5)	32.2 (29.7, 35.1)
1st/2nd/3rd trimester (n)	–	20/78/79
% taking folate supplements	11.1	82.5
Red blood cell folate (nmol/L) ^1^	442 (366, 564)	873 (677, 1177)
% RBC folate <340 nmol/L	19.9 (*n* = 34)	2.8 (*n* = 5)
% RBC folate <906 nmol/L	91.8 (*n* = 157)	52.0 (*n* = 92)

^1^ Median (interquartile range), RBC: red blood cell.

**Table 2:** Participant characteristics in the survey on folate knowledge in Switzerland (*n* = 784).

	Total		Women		Men	
	*n*	%	*n*	%	*n*	%
**Total**	784	100	482	61	302	39
**Nationality**						
Swiss	634	81	385	80	249	82
Non–Swiss	150	19	97	20	53	18
**Education**						
No school degree	4	0.5	3	0.6	1	0.3
No tertiary degree	148	19	66	14	82	27
Tertiary degree	632	81	413	86	219	73
**Monthly income**						
No answer	85	11	52	11	33	11
<CHF 6000	175	22	111	23	64	21
≥CHF 6000	524	67	319	66	205	68
**Diet**						
Special diet *	153	20	102	21	51	17
No special diet	631	80	380	79	251	83
**Nutritional awareness**						
Low	191	24	64	13	127	42
Medium	352	45	218	45	134	44
High	241	31	200	42	41	14

* At the time of the study, participants were (self–declared) vegetarian, vegan, pescetarian or other.

## Supplementary Materials

The following are available online at https://www.mdpi.com/2072-6643/12/12/3729/s1. Table S1, Supplementary material: Survey questionnaire for a study on folate knowledge in the Swiss population.
