# Supplementary material for: Inadequate Status and Low Awareness of Folate in Switzerland—A Call to Strengthen Public Health Measures to Ensure Sufficient Intakes
Source: Nutrients. 2020 Dec 3;12(12):3729. doi: 10.3390/nu12123729 (PMC7761771; doi:10.3390/nu12123729)
Supplement: Supplementary file 1 [file nutrients-12-03729-s001.pdf]

Supplementary material

Table S1 Survey questionnaire for a study on folate knowledge in the Swiss population

Question 1

What is folic acid? (only one answer possible)

	An essential vitamin
	A mineral
	A food acid (as e.g. citric acid)
	A corrosive acid
	Don't know

Question 2

Which foods are naturally rich in folic acid? (multiple answers possible)

	Vegetables
	Fruit
	Pulses
	Wheat germ
	eggs
	Meat (excl. offal)
	Milk / dairy
	Don't know

Question 3

Please assess the following statements (correct / wrong):

	Correct	Wrong
Folic acid has a positive influence on the sperm amount and quality in men.		
Folic acid reduces the risk of an "open back" (=spina bifida) and other birth defects.		
Folic acid reduces the risk of bone fractures in the older age (=Osteoporosis).		
Folic acid enhances the absorption of vitamin C from food.		
Folic acid is important for the normal development of a child in the womb.		
Folic acid supports the prevention of cardiovascular diseases.		
Folic acid is important for healthy teeth.		
Folic acid has a positive influence on the capacity of memory in the older age.		

Supplemental table 1

Question 4

What is the correct recommendation for the intake of folic acid supplements to reduce the risk of an open back (=spina bifida) in a new-born? (only one answer possible)

	Men: at least 4 weeks ahead of procreation
	Women: at least 4 weeks ahead of procreation and during the first 12 weeks of pregnancy
	Women: after one learns about the pregnancy
	Women: during lactation
	Babies: with baby food
	Don't know

Question 5

Please rate the following statements on a scale of 1 (I don't agree at all) to 5 (I fully agree).

	I don't agree at all				I fully agree
I am interested in the topics nutrition and health					
I am interested in the topic folic acid.					
I pay attention to a healthy and balanced diet					
I consciously pay attention to having a sufficient intake of folic acid.					

Question 6

Do you follow a specific diet and if so, which one?

	Vegetarian
	Vegan
	Pescetarian (no meat, but I eat fish)
	Omnivore (I eat everything)
	Other: please specify

Question 7

How often do you use the following information channels to obtain information about nutrition and health?

	Never				Very often
Doctor / medical professionals					
Pharmacy					
Media (offline and online, incl. social media)					
Friends / family					
School / educational establishment					
Specialist literature and media (e.g. website of the folic acid foundation Switzerland)					
Other: please specify					

Supplemental table 1

Question 8

How would you rate the reliability of the following information channels regarding nutrition and health?

	Not at all reliable				Highly reliable
Doctor / medical professionals					
Pharmacy					
Media (offline and online, incl. social media)					
Friends / family					
School / educational establishment					
Specialist literature and media (e.g. website of the folic acid foundation Switzerland)					
Other: please specify					

Question 9

Where do you mainly have your current knowledge about folic acid from? (only one answer possible)

	Doctor / medical professionals
	Pharmacy
	Media (offline and online, incl. social media)
	Friends / family
	School / educational establishment
	Specialist literature and media (e.g. website of the folic acid foundation Switzerland)
	Other: please specify
	I don't know anything about folic acid.

Question 10

Which is your preferred information channel regarding information about folic acid? (multiple answers possible)

	Doctor / medical professionals
	Pharmacy
	Media (offline and online, incl. social media)
	Friends / family
	School / educational establishment
	Specialist literature and media (e.g. website of the folic acid foundation Switzerland)
	Other: please specify

Question 11

Please indicate your year of birth:

	Free entry of date or drop-down

Question 12

Please indicate your sex:

	Male
	Female

Supplemental table 1

Question 13

Please indicate your nationality? (multiple answers possible)

	Free entry
	Free entry
	Free entry

Question 14

What is the highest educational degree you obtained?

	No school degree
	Obligatory school (primary school) completed
	Secondary school: apprenticeship, commercial matura or high-school matura
	Degree of a University of Applied Sciences
	Degree of a University / ETH
	Doctorate

Question 15

What is the monthly net income of your household approximately?

	Less than 3'000 CHF
	Between 3'000 and 4'499 CHF
	Between 4'500 and 5'999 CHF
	Between 6'000 and 8’999 CHF
	Between 9'000 and 12'999 CHF
	More than 13'000 CHF
	No answer

Question 16

Please indicate the zip code of your domicile:

	Free entry

End of survey for all male participants.

In case question 15 was answered with „female“:

Question 17

Do you regularly (every 1-2 years) visit a gynaecologist for a check-up?

	Yes
	No

Question 18

Has your gynaecologist informed you about the necessity to take folic acid supplements in connection with a pregnancy **in a regular check-up?**

	Yes
	No

Supplemental table 1

Question 19

Are you pregnant?

	Yes
	No

Question 20

Do you have children?

	Yes
	No

In case question 19 or question 20 was answered with „yes", questions 24 and 25 are asked. Otherwise, the survey terminates here.

Question 21

At which point in time during your pregnancy / pregnancies have you started taking folic acid supplements:

	Earlier than 1 month ahead of pregnancy
	During the first 4 weeks of pregnancy
	Between the 5-8. week of pregnancy
	Between the 9-12. week of pregnancy
	Upon the 13. week of pregnancy
	I did / do not take any folic acid supplements

Question 22

Has your gynaecologist informed you about the following topics prior or during your pregnancy?

	Yes, prior to pregnancy	Yes, during pregnancy	No
Nutrition during pregnancy.			
Necessity to take folic acid supplements in connection with a pregnancy.			
The connection between the intake of folic acid and the clinical picture of "open back" (=spina bifida).			
The connection between the intake of folic acid and other birth defects.			
